# High Expression of Hyaluronan-Mediated Motility Receptor Predicts Adverse Outcomes: A Potential Therapeutic Target for Head and Neck Squamous Cell Carcinoma

**DOI:** 10.3389/fonc.2021.608842

**Published:** 2021-03-08

**Authors:** Tianzhu Lu, Yahan Zheng, Xiaochang Gong, Qiaoli Lv, Junjun Chen, Ziwei Tu, Shaojun Lin, Jianji Pan, Qiaojuan Guo, Jingao Li

**Affiliations:** ^1^Department of Radiation Oncology, Jiangxi Cancer Hospital of Nanchang University, Nanchang, China; ^2^National Health Commission (NHC) Key Laboratory of Personalized Diagnosis and Treatment of Nasopharyngeal Carcinoma (Jiangxi Cancer Hospital of Nanchang University), Nanchang, China; ^3^Department of Radiation Oncology, Fujian Cancer Hospital and Fujian Medical University Cancer Hospital, Fuzhou, China; ^4^Fujian Provincial Key Laboratory of Translational Cancer Medicine, Fujian Cancer Hospital, Fuzhou, China

**Keywords:** head and neck squamous cell carcinoma, HMMR, prognosis, biomarker, biological functions

## Abstract

**Background:** Several studies have shown that the hyaluronan-mediated motility receptor (HMMR) is overexpressed in various cancers and could be a potential prognostic factor. However, further research is still required to determine the prognostic value and potential function of HMMR in head and neck squamous cell carcinoma (HNSCC).

**Materials and Methods:** Transcriptomic expression data were collected from the Cancer Genome Atlas database (TCGA) and Gene Expression Omnibus and the differences in HMMR expression between normal and tumor tissues were analyzed. The correlation between the methylation level of HMMR and its mRNA expression was analyzed via cBioPortal. Additionally, the data obtained from TCGA was analyzed with MethSurv to determine the prognostic value of the HMMR methylation levels in HNSCC. Gene set enrichment analysis (GSEA) and single sample GSEA (ssGSEA) were used to explore the potential biological functions of HMMR.

**Results:** HMMR was highly expressed in HNSCC tumor tissue compared to normal tissue (*p* < 0.001). Multivariate analysis (MAV) showed that high HMMR mRNA expression was an independent prognostic factor of overall survival (OS) in TCGA (HR = 1.628, 95% CI: 1.169–2.266, *p* = 0.004) and GSE41613 data (HR = 2.238, *p* = 0.013). The methylation level of HMMR negatively correlated with the HMMR expression (*R* = −0.12, *p* < 0.001), and patients with low HMMR methylation had worse OS than patients with high methylation (*p* < 0.001). GSEA found that HMMR expression was associated with the KARS, EMT, and G2M checkpoint pathways, as well as the interferon-gamma and interferon-alpha responses, whereas ssGSEA showed that *HMMR* expression positively correlated with the infiltration level of Th2 cells. MAV confirmed that high HMMR protein expression was an inferior independent factor for OS (HR = 2.288, *p* = 0.045) and progression-free survival (HR = 2.247, *p* = 0.038) in 70 HNSCC.

**Conclusions:** This study demonstrated that the upregulation of HMMR mRNA and protein in HNSCC is a biomarker for poor prognosis. The biological functions of HMMR are potentially related to the KARS, EMT, and G2M checkpoint pathways, as well as the interferon-gamma and interferon-alpha responses. These findings help to elucidate the role of HMMR in carcinogenesis and lay a foundation for further study.

## Introduction

Head and neck squamous cell carcinoma (HNSCC) is a common malignancy, with 500,000 new cases occurring every year worldwide (1, 2). As most patients with HNSCC are diagnosed in the late stages, often with metastasis, the 5-year overall survival (OS) rate is only around 50% (3, 4). For the standard management of HNSCC, the TNM classification system uses the tumor size, location, and metastatic state to evaluate a patient's prognosis; based on this, a treatment strategy is then developed (5). However, this system has several flaws as patients with the same TNM stage respond differently to treatment (6).

It is important to find stable and reliable tumor markers to screen patients for poor prognosis and provide more aggressive treatment. HNSCC is a highly heterogeneous disease (7). Firstly, the main reasons for this are: its diverse origins as HNSCC arises from the upper aerodigestive tract epithelia, which includes the oral cavity, oropharynx, and hypopharynx. Secondly, some tumors are a subtype that is closely related to the human papillomavirus (HPV), and these have a significantly better prognosis (7, 8). These heterogeneities are the reason behind the need for stable, reliable, and broad-spectrum biomarkers, on the other hand, they are also the obstacles against finding stable biomarkers.

Glycosaminoglycan hyaluronic acid (HA) is a ubiquitous component of the extracellular matrix (ECM) and is highly expressed in the tumor microenvironment (9–11). Overexpression of HA can potentially promote tumor growth and metastasis. There are two cell membrane receptors associated with HA, namely *CD44* and *HMMR* (hyaluronan-mediated motility receptor). The biological function of *CD44* is relatively well-known (12), but the biological function of *HMMR* is not yet fully understood. Many studies have found that *HMMR* is highly expressed in various malignant tumors, including bladder cancer (13), pancreatic cancer (14, 15), glioma (16), gastric cancer (17), and colorectal cancer (18) and so on. Some studies showed that a high expression of *HMMR* is associated with worse prognosis as it promotes cancer growth and metastasis. Consideration of HMMR overexpressed in various cancer types, *HMMR* was considered a tumor-associated antigen and therapeutic target of immunotherapy. Previous studies have shown that short peptides of *HMMR* can be effectively presented by DC cells and activate T cell immunity (19–22). Besides, T-cell receptor-engineered T-cell therapy (TCR-T) for *HMMR* efficiently inhibitor tumor growth in an animal model (23). Although some researchers found that HMMR was overexpression and a potential biomarker for HNSCC (24–26), the prognostic value and the potential function of *HMMR* in HNSCC were unclear and not confirmed. Thus, it was worth in-depth study the prognostic value and the potential function of HMMR in HNSCC.

This study comprehensively evaluated the prognostic value of *HMMR* mRNA expression and methylation in patients with HNSCC using data from the Cancer Genome Atlas (TCGA) database. Furthermore, the prognostic value of *HMMR* mRNA expression in HNSCC was validated using data from the Gene Expression Omnibus (GEO) databases. Additionally, we also performed GSEA analysis to gain further insights into the biological role of *HMMR* in HNSCC pathogenesis. Furthermore, we explored the *HMMR* expressionin HNSCC cell lines and tissues, and the prognostic value of HMMR protein detected by immunohistochemistry (IHC) in 70 HNSCC patients.

## Materials and Methods

### Data Acquisition

This study includes two data sets from the TCGA database (https://www.cancer.gov/about-nci/organization/ccg/research/structural-genomics/tcga), the RNA-seq transcriptomic data and the corresponding patient clinical data from HNSCC samples. RNA sequence data from 528 patients with HNSCC and 44 normal tissues were downloaded from TCGA database (https://portal.gdc.cancer.gov). The RNA-seq data and the patient clinical information (Workflow Type: HTSeq-FPKM) were acquired using the Data Transfer Tool (provided by GDC Apps). Subsequent data processing excluded cases without survival data, and the remaining data (*n* = 500) is shown in Supplementary Table 1. The level 3 HTSeq-FPKM data were transformed to TPM (transcription per million reads) for the following analyses. Patients with HNSCC were classified into low- and high-expression groups according to their median expression value of *HMMR*. The study used R to download the *HMMR* mRNA expression data and clinical data in the GSE41613 data form from the GEO databases as an external validation of survival analyses.

### Analysis of Differentially Expressed Genes (DEGs) Between the High and Low *HMMR* Expression Groups in Patients With HNSCC

Expression profiles (HTSeq-TPM) were compared between the high and low *HMMR* mRNA expression groups to identify the DEGs using the unpaired Student's *t*-test, within the limma Package software (27). A |log2Fold Change| > 1.5 and adjusted *P* < 0.001 were considered the threshold for the DEGs.

### Gene Ontology (GO) Enrichment Analysis

Metascape (https://metascape.org) is a tool for gene annotation and pathway analysis (28). In this study, Metascape was used to analyze the enrichment of HMMR related DEGs by process and pathway. The GO terms for biological process, cellular component, and molecular function categories were enriched based on the Metascape online tool. Only terms with a *P* < 0.01, a minimum count of 3, and an enrichment factor of >1.5 were considered as significant.

### Gene Set Enrichment Analysis (GSEA)

GSEA is an analytical method that determines whether a previously defined set of genes shows statistically significant, concordant differences between two phenotypes (29). In this study, GSEA was carried out using the R package clusterProfiler (3.8.0) (30) in order to elucidate the significant function and pathway differences between the high- and low-*HMMR* groups. Gene set permutations were performed 1,000 times for each analysis. The expression level of *HMMR* mRNA was used as a phenotype label. The study chose h.all.v7.0.symbols.gmt [Hallmarks] in the MSigDB Collections as the reference gene collection. An adjusted *P* < 0.05, False discovery rate (FDR) < 0.25, and normalized enrichment score (|NES|) > 1 were considered as significant enrichment.

### Analysis of Immune Infiltration and Its Correlation With HMMR Expression

By applying the ssGSEA (single-sample Gene Set Enrichment Analysis) method from the GSVA package (31) in R, we quantified the relative tumor infiltration levels of immune cell types by integrating the expression levels of genes in published signature gene lists (32). To evaluate the association between the infiltration of immune cells and the different HMMR mRNA expression groups, the Wilcoxon rank-sum test and Pearson correlation were carried out. TIMER software (33) was used to validate the correlation between the different *HMMR* mRNA expression levels and the infiltration of immune cells in HNSCC samples from TCGA database.

### HMMR Methylation Level and Its Prognosis Analysis

The copy number variation (CNV) and methylation level data of *HMMR* were obtained through the cBioPortal web platform (https://www.cbioportal.org/) and a comparison of the varying *HMMR* gene expressions in *HMMR* copy number variation groups (Kruskal–Wallis test) and the correlation between *HMMR* methylation level and *HMMR* gene expression (Person correlation) was conducted. SMART web platform (http://www.bioinfo-zs.com/smartapp/) was used to analyze and compare the methylation levels of *HMMR* in pan-cancer and normal tissues from TCGA data. The UALCAN online tool (http://ualcan.path.uab.edu/) was used to analyze the differences in the expressions of *HMMR* mRNA in HNSCC and normal tissues from TCGA data. MethSurv online tool (https://biit.cs.ut.ee/methsurv/) was used to analyze the prognostic value of the *HMMR* methylation level in HNSCC (TCGA data).

### Prognostic Model Generation and Prediction

Multivariate Cox regression analysis and Akaike's information criterion (AIC) method were used to determine the optimal prognostic model. Additionally, a nomogram was constructed to predict the prognosis by R packages rms. The patients were stratified into a high- and low-risk groups based on the median value of their risk scores. The difference in OS between the high-risk group and low-risk group were determined by the Kaplan–Meier method with a two-sided log-rank test. Receiver operating characteristic (ROC) curve was constructed to evaluate the prediction accuracy of the prognostic model intensity.

### Tissue Specimens, and Cell Lines, and Culture

Twenty-four samples from patients with HNSCC who received no treatment from January 2019 to December 2019 in Jiangxi Cancer Hospital of Nanchang University were selected, and 12 para-carcinoma tissue from January 2019 to December 2019 were included. Tissue samples were stored in liquid nitrogen for transport and long-term storage to prevent RNA degradation. Twenty-four fresh-frozen tissue samples were diagnosed by histopathology and approved for use by the hospital ethics committee.

Three HNSCC cell lines (CAL27, SCC9, and FaDu) were purchased from the American Type Culture Collection (ATCC). Moreover, one normal human oral keratinocyte (HOK) cell line was purchased from ScienCell research laboratories, and was cultivated in oral keratinocyte medium (OKM, Cat. 2611, ScienCell). CAL27 was sustained in DMEM (Cat. L110KJ, BasaMedia, Shanghai, China) with 10% FBS, FaDu was sustained MEM (Cat. L550KJ, BasaMedia, Shanghai, China) with 10% FBS, and SCC-9 cells were grown in 1:1 Hams F-12, DMEM (Cat. L310KJ, BasaMedia, Shanghai, China). All cell lines were authenticated by short tandem repeat analysis.

### RNA Isolation and qRT-PCR

Total RNA was extracted from tissues using TRIzol reagent (TaKaRa, Tokyo, Japan) based on the manufacturer's protocol. The cDNA was transcribed using the PrimeScript RT Reagent Kit (Cat. RR047A, TaKaRa). The SYBR Green PCR Kit (Cat. RR820A, Takara) was used to detect isolated RNA quantity. qRT-PCR was performed using the CFX Connect Real-Time System (No. 788BR07388, Bio-Rad, USA). PCR amplification was performed as follows: 95.0°C for 3 min, and 40 circles of 95.0°C for 10 s, and 60°C for 30 s. The primers for *HMMR* mRNA C1S were F: 5′-CAGGCCTTAGAAGCTGACATGAGC-3′, and R: 5′-TCCAAACTTCTCACTGCAGACAGC-3′. GAPDH: F: 5′-CCCATCACCATCTTCCAGGAG-3′, R: 5′-GTTGTCATGGATGACCTTGGC-3′.

### Immunohistochemistry (IHC)

Tissue samples from 70 HNSCC cases with diagnosed pathology treated at the Jiangxi Cancer Hospital from 2013 to 2017 were included in the analysis. All patients with HNSCC were classified according to the TNM stage classification scheme (7th edition, AJCC). This study was approved by the Hospital Review Board of Jiangxi Cancer Hospital (No. 202000137), Jiangxi, China. The samples were fixed in formaldehyde and processed with heat-mediated antigen retrieval in citrate buffer (PH = 6). The samples were then blocked and incubated with rabbit polyclonal anti-HMMR (1:250, ab124729, Abcam, USA) at 4°C overnight. ElivisionTM plus Polyer HP (Mouse/Rabbit) IHC Kit (Cat. KIT-9901, MXB biotechnologies, China) was used. Two independent pathologists, who were blinded to the clinical outcome, evaluated staining. According to the staining intensity, HMMR protein expression was divided into negative (**Figure 7C**), weakly (**Figure 7D**), moderately (**Figure 7E**), and strongly positive (**Figure 7F**), as shown in **Figure 7**.

### Statistical Analyses

The Wilcoxon rank-sum test and Wilcoxon signed-rank test were used to analyze the expression of HMMR in non-paired and paired samples, respectively. The ROC curve was generated to evaluate the diagnostic performance of HMMR expression using the pROC package. The Kruskal–Wallis test, Wilcoxon signed-rank test, and Chi-Squared test were used to analyze the relations between the clinicopathological features and HMMR expression. Survival curves were drawn using the Kaplan–Meier method, and the differences between groups were assessed via the log-rank test. Univariate and multivariate analyses using Cox proportional hazard modeling were performed to estimate the risk of death. Potential confounders included gender, age, clinical stage, and treatment and so on. A *P* < 0.05 (two-sided) was considered statistically significant. Statistical analyses were carried out using R (version 3.6.1) and SPSS (version 24.0).

## Results

### HMMR Was Upregulated in HNSCC

**Figure 1:**
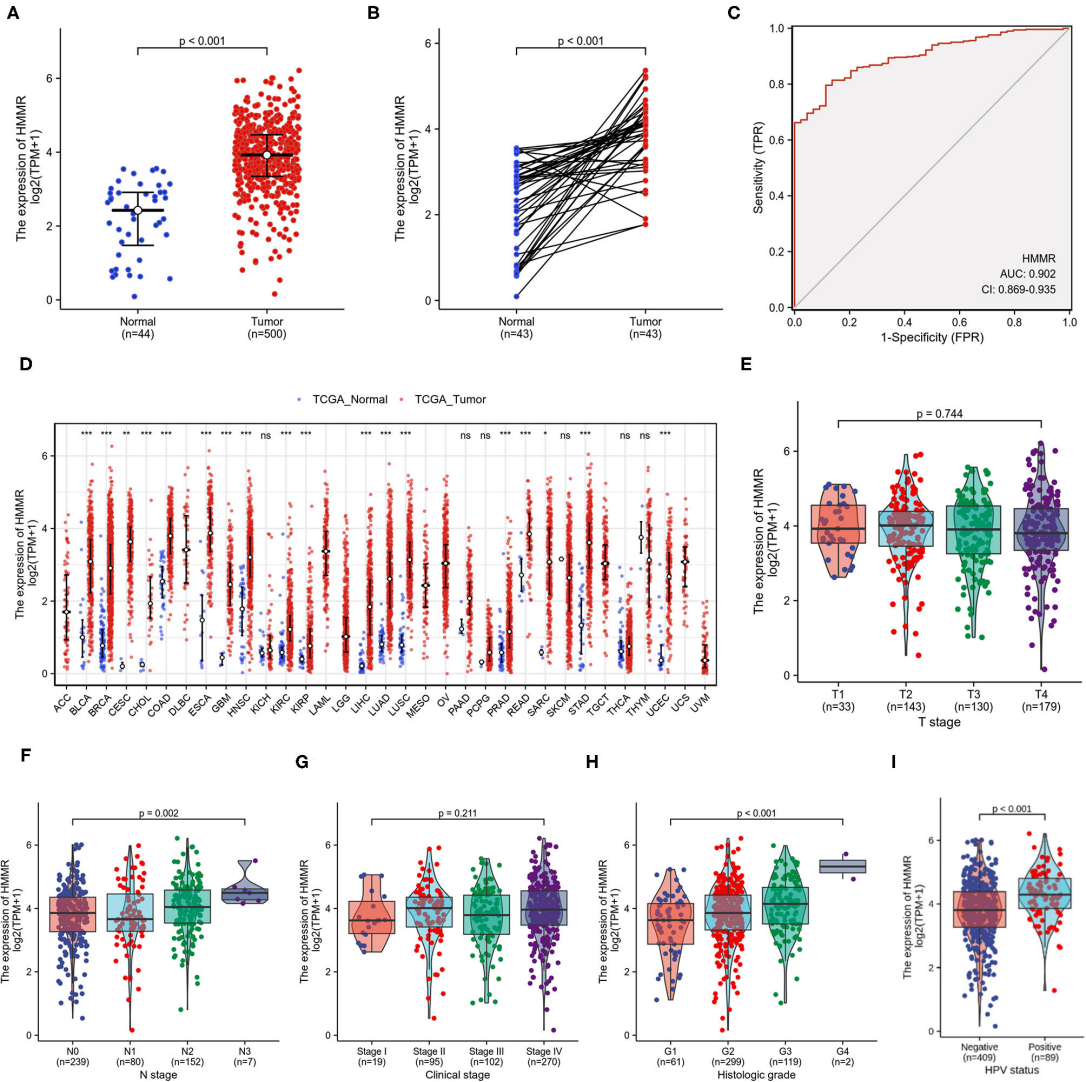
HMMR mRNA in HNSCC and other types of human cancers from TCGA data. **(A)** Expression levels of HMMR in HNSCC (*n* = 500) and normal tissue (*n* = 44); **(B)** The expression of HMMR in HNSCC (*n* = 43) and its paired adjacent tissues (*n* = 43); **(C)** Receiver operating characteristic analysis (ROC) of HMMR in HNSCC (*n* = 544); **(D)** HMMR expression levels in different tumor types from TCGA database; **(E)** The association of HMMR expression and T classification in HNSCC (*n* = 485); **(F)** The association of HMMR expression and N classification in HNSCC (*n* = 478); **(G)** The association of HMMR expression and clinical stages in HNSCC (*n* = 486); **(H)** The association of HMMR expression and histologic grade (*n* = 481); **(I)** The HMMR expression in HPV positive and negative HNSCC (*n* = 498) (^*^P < 0.05, ^**^P < 0.01, and ^***^P < 0.001).

The results showed that HMMR was highly expressed in HNSCC tumor tissue compared with normal tissue (*p* < 0.001; Figure 1A). In paired specimens, the expression of *HMMR* mRNA in the HNSCC group was significantly higher than that found in the adjacent normal tissues (*p* < 0.001) (Figure 1B). ROC showed that the expression of *HMMR* mRNA in HNSCC was 0.902 (95% CI: 0.869–0.935) (Figure 1C) and the best cut-off value of HMMR was 8.337 (TPM).

To further evaluate *HMMR* mRNA expression in human cancers, we examined *HMMR* mRNA expression using the pan-cancer RNA-seq data from TCGA. The differential expression of *HMMR* mRNA between the tumor and adjacent normal tissues is shown in Figure 1D. *HMMR* mRNA expression was significantly overexpressed in almost all tumor types in TCGA database compared to the normal tissue, including urothelial bladder carcinoma (BLCA), invasive breast carcinoma (BRCA), cervical squamous cell carcinoma and endocervical adenocarcinoma (CESC), cholangiocarcinoma (CHOL), colon adenocarcinoma (COAD), esophageal carcinoma (ESCA), glioblastoma multiforme (GBM), head-neck squamous cell carcinoma (HNSC), kidney renal clear cell carcinoma (KIRC), kidney renal papillary cell carcinoma (KRPA), hepatocellular carcinoma (LIHC), lung adenocarcinoma (LUAD), lung squamous cell carcinoma (LUSC), prostate adenocarcinoma (PRAD), rectum adenocarcinoma (READ), stomach adenocarcinoma (STAD), and uterine corpus endometrial carcinoma (UCEC).

The *HMMR* mRNA expression was strongly associated with N classification (Figure 1F) and high histological grade (Figure 1H), whereas no associations were detected with T classification (Figure 1E) or clinical stage (Figure 1G). The results showed that the *HMMR* mRNA expression in HPV-positive patients was higher than that in HPV-negative patients (Figure 1I). Other clinical characteristics did not correlate with the expression of *HMMR*; detailed information on this is shown in Table 1.

**Table 1:** Demographic and clinical characteristics of HNSCC patients with low- and high expression HMMR in TCGA (*n* = 500).

**Characters**	**Level**	**HMMR expression**		***p***
		**Low (*n* = 250)**	**High (*n*−250)**	
Gender (%)	Female	70 (28.0%)	63 (25.2%)	0.544
	Male	180 (72.0%)	187 (74.8%)	
Age (median [IQR])		62.0 [54.3,69.0]	60.0 [53.0,68.0]	0.237
T stage	T1	16 (6.4%)	17 (6.8%)	0.241
	T2	62 (24.8%)	81 (32.4%)	
	T3	66 (26.4%)	64 (25.6%)	
	T4	98 (39.2%)	81 (32.4%)	
	Missing	8 (3.2%)	7 (2.8%)	
N stage	N0	126 (50.4%)	113 (45.2%)	<0.001
	N1	50 (20.0%)	30 (12.0%)	
	N2	62 (24.8%)	90 (36.0%)	
	N3	0 (0.0%)	7 (2.8%)	
	Missing	12 (4.8%)	10 (4%)	
M stage	M0	233 (93.2%)	237 (94.8%)	0.684
	M1	3 (1.2%)	2 (0.8%)	
	Missing	14 (5.6%)	11 (4.4%)	
Clinical stage	Stage I	13 (5.2%)	6 (2.4%)	0.045
	Stage II	41 (16.4%)	54 (21.6%)	
	Stage III	60 (24.0%)	42 (16.8%)	
	Stage IV	129 (516%)	141 (56.4%)	
	Missing	5 (2%)	7 (2.8%)	
Histologic grade	G1	40 (16.3%)	21 (8.4%)	0.035
	G2	157 (64.1%)	142 (56.8%)	
	G3/G4	48 (19.6%)	73 (29.2%)	
	Missing	5 (2.0%)	14 (5.6%)	
Radiation therapy	No	73 (29.2%)	80 (32.0%)	0.360
	Yes	151 (60.4%)	135 (54.0%)	
	Missing	26 (10.4)	35 (14.0%)	
Anatomic site	Oral cavity	167 (66.8%)	141 (56.4%)	<0.001
	Oropharynx	17 (6.8%)	54 (21.6%)	
	Hypopharynx	6 (2.4%)	4 (1.6%)	
	Larynx	60 (24.0%)	51 (20.4%)	
Smoker	No	54 (21.6%)	57 (22.8%)	0.829
	Yes	191 (76.4%)	188 (75.2%)	
	Missing	5 (2.0%)	5 (2.0%)	
Alcohol history	No	86 (35.0%)	71 (28.4%)	0.207
	Yes	160 (65.0%)	172 (68.8%)	
	Missing	4 (1.6%)	7 (2.8%)	
Race	Asian	6 (2.4%)	4 (1.6%)	0.811
	Black or African	23 (9.2%)	24 (9.6%)	
	White	213 (85.2%)	213 (85.2%)	
	Missing	8 (3.2%)	9 (3.6%)	
TP53 status	Mut	173 (69.2%)	161 (64.4%)	0.354
	WT	75 (30.0%)	85 (33.6%)	
	Missing	2 (0.8%)	4 (1.6%)	
PIK3CA status	Mut	42 (16.8%)	42 (16.8%)	1.000
	WT	206 (82.4%)	204 (81.6%)	
	Missing	2 (0.8%)	4 (1.6%)	
HPV status	Negative	223 (89.2%)	186 (74.4%)	<0.001
	Positive	25 (10.0%)	64 (25.6%)	
	Missing	2 (0.8%)	NA	

*HNSCC, Head and neck squamous cell carcinoma; TCGA, The Cancer Genome Atlas database; HMMR, hyaluronan-mediated motility receptor*.

### High HMMR Expression Is Associated With Adverse Outcomes in HNSCC

**Figure 2:**
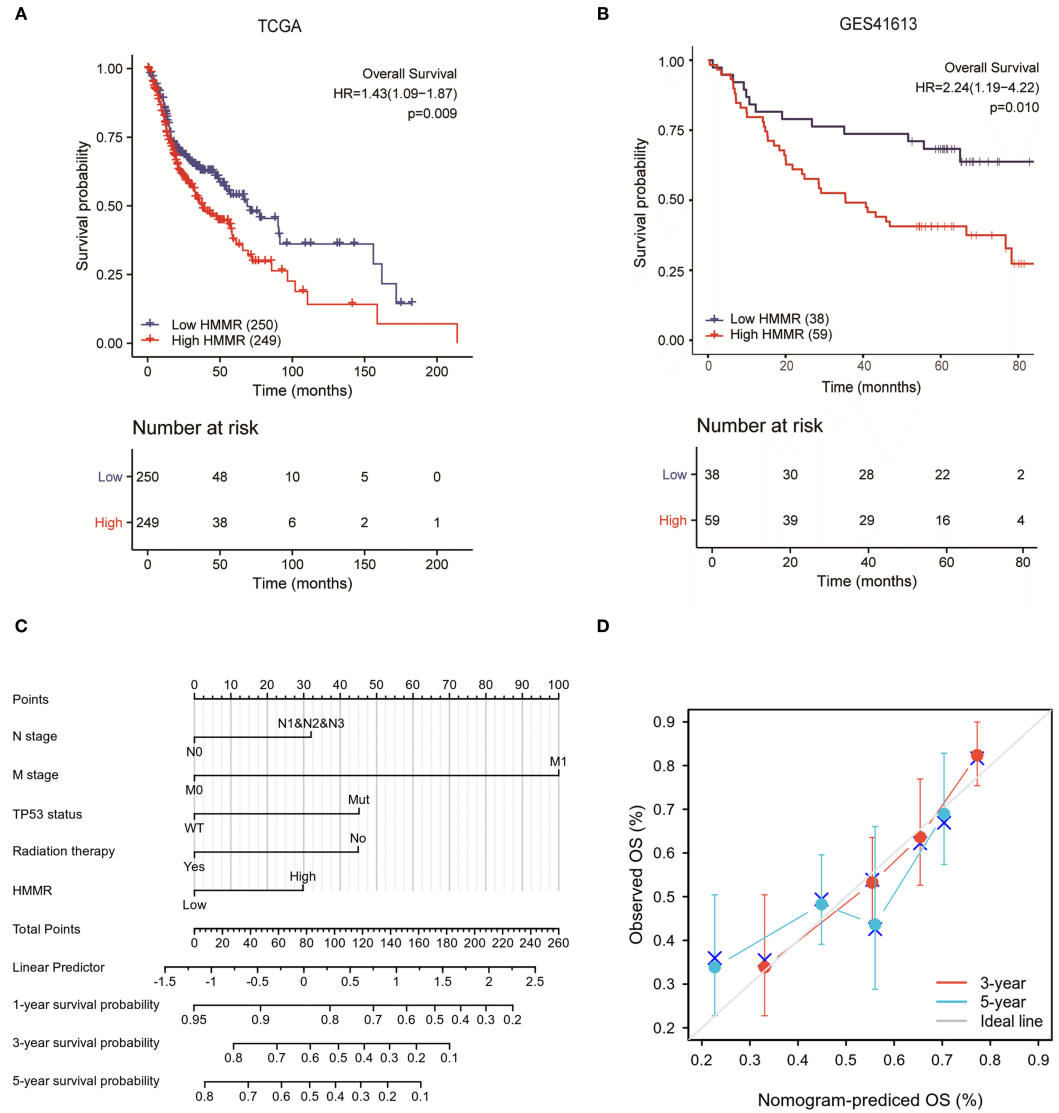
The prognostic value of HMMR expression in HNSCC. **(A)** Survival curves of OS from TCGA data (*n* = 500); **(B)** Survival curves of OS from GSE 41613 data (*n* = 97); **(C)** A nomogram that integrates HMMR and other prognostic factors in HNSCC from TCGA data; **(D)** The calibration curve of the nomogram.

There were 367 male and 133 female patients with a median age of 62 years old (interquartile range from 54 to 68). The expression level of *HMMR* mRNA in HNSCC was classified as low- or high-expression according to the median value (14.173 for TPM). Detailed clinicopathological features are shown in Table 1. The Kaplan–Meier survival analysis showed that patients with high HMMR expression in the TCGA-HNSCC data set had a worse OS than patients in the low expression group (HR = 1.432, 95% CI: 1.094–1.875, *p* = 0.009; Figure 2A). Multivariate analysis also showed that *HMMR* mRNA expression is an independent prognostic factor of OS for HNSCC (HR = 1.628 95%, CI: 1.169–2.266, *p* = 0.004). In addition, N stage (HR = 1.606, 95% CI: 1.141–2.259, *p* = 0.007), M stage (HR = 4.037, 95% CI: 1.126–14.476, *p* = 0.032), TP53 status (HR = 1.948, 95% CI: 1.343–2.825, *p* < 0.001) and Radiation therapy (HR = 0.502, 95% CI: 0.628–0.710, *p* < 0.001) are also independent prognostic factors (Table 2) of OS. The Kaplan–Meier analysis showed that patients with high *HMMR* mRNA expression in the TCGA-HNSCC had a worse PFS than patients in the low expression group (HR = 1.470, 95% CI: 1.110–1.960, *p* = 0.008) (Supplementary Figure 1). Multivariate analysis also showed that *HMMR* mRNA expression is an independent prognostic factor of PFS for HNSCC (HR = 1.453, 95% CI: 1.086–1.943, *p* = 0.012) (Supplementary Table 1). Considering that HPV-positive oropharyngeal cancer (OPC) is a type of virus-related tumor with a better prognosis, we further analyzed the prognosis of HMMR in HPV-positive OPC and HNSCC excepted for HPV-positive OPC. The results showed that in the HNSC excepted for HPV-positive OPC, the OS of patients with high HMMR expression was significantly worse than that of patients with low HMMR expression (*p* = 0.0039; Supplementary Figure 2A). In HPV-positive OPC, the OS of patients with high- and low-HMMR expression was similar, and the difference was not statistically significant (*p* = 0.82; Supplementary Figure 2B).

**Table 2:** The univariate and multivariate analyses of overall survival according to HMMR expression, after adjusting for other potential predictors in TCGA (*n* = 500).

**Characteristics**	**Univariate analysis**		**Multivariate analysis**	
	**HR (95% CI)**	***p-*value**	**HR (95% CI)**	***p-*value**
T stage (T1–2 vs. T3–4)	1.230 (0.921–1.642)	0.160		
N stage (N0 vs. N1–3)	1.257 (0.960–1.647)	0.097	1.606 (1.141–2.259)	0.007
M stage (M1 vs. M0)	4.794 (1.765–13.016)	0.002	4.037 (1.126–14.476)	0.032
Clinical stage (I–II vs. III–IV)	0.824 (0.594–1.142)	0.245		
Age (≤ 60 vs. >60)	1.238 (0.945–1.621)	0.122		
Gender (Female vs. Male)	0.754 (0.566–1.004)	0.054	0.794 (0.561–1.124)	0.194
Histologic grade (G1–2 vs. G3–4)	0.942 (0.690–1.287)	0.709		
Smoker (No vs. Yes)	1.085 (0.775–1.520)	0.633		
Alcohol history (No vs. Yes)	0.967 (0.727–1.288)	0.821		
TP53 status (WT vs. Mut)	1.531 (1.119–2.094)	0.008	1.948 (1.343–2.825)	<0.001
PIK3CA status (WT vs. Mut)	0.988 (0.702–1.392)	0.946		
Radiation therapy (No vs. Yes)	0.623 (0.459–0.846)	0.002	0.502 (0.355–0.710)	<0.001
Race (White vs. Non–white)	1.477 (0.977–2.231)	0.064	1.055 (0.628–1.775)	0.839
HPV (Negative vs. Positive)	1.335 (0.894–1.995)	0.158		
HMMR (Low vs. High)	1.432 (1.094–1.875)	0.009	1.628 (1.169–2.266)	0.004

*TCGA, The Cancer Genome Atlas database; HMMR, hyaluronan-mediated motility receptor*.

To further verify the prognostic value of *HMMR* mRNA expression in HNSCC, we included the GSE41613 data set from GEO along with the survival data. According to the method with the smallest *p*-value in the Kalan–Meier method, the cut-off value of *HMMR* mRNA expression is 2.08578. Survival analysis showed that the high HMMR had a worse OS (HR = 2.240, 95% CI: 1.189–4.221, *p* = 0.013) (Figure 2B). Multivariate analysis using GSE41613 data showed that *HMMR* mRNA is also an independent prognostic factor for HNSCC (HR = 2.238, 95% CI: 1.187–4.221, *p* = 0.013) (Table 3).

**Table 3:** The univariate and multivariate analyses of overall survival according to HMMR expression, after adjusting for other potential predictors in GSE41613 (*n* = 97).

**Characteristics**	**Univariate analysis**		**Multivariate analysis**	
	**HR (95% CI)**	***P*-value**	**HR (95% CI)**	***P-*value**
Clinical stage (I–II vs. III–IV)	3.828 (1.958–7.482)	<0.001	6.078 (2.603–14.919)	<0.001
Age (≤ 60 vs. >60)	0.739 (0.423–1.292)	0.289		
Gender (Female vs. Male)	0.891 (0.493–1.611)	0.703		
Comprehensive treatment (No vs. Yes)	1.671 (0.939–2.974)	0.081	0.532 (0.256–1.104)	0.090
HMMR (Low vs. High)	2.240 (1.189–4.221)	0.013	2.238 (1.187–4.221)	0.013

*HMMR, hyaluronan-mediated motility receptor*.

### Establishment of the Prognostic Models for HNSCC

The results mentioned above suggest that *HMMR* mRNA is an independent prognostic factor in HNSCC. To verify this, we established a prediction model for OS and PFS by fitting HMMR mRNA expression and other clinicopathological parameters from the TCGA data. We constructed a nomogram of OS to integrate *HMMR* and other prognostic factors, including N classification, M classification, TP53 mutation, and radiation therapy (Figure 2C). A higher point on the nomogram represented a worse prognostic factor. The Calibration curve evaluated the nomogram's performance of *HMMR*, and the C-index of OS was 0.715 (Figure 2D). In summary, this nomogram may be a better model for predicting survival in patients with HNSCC than individual prognostic factors.

### Hypomethylation Correlates With the Expression of *HMMR* mRNA and Indicates an Adverse Outcome in HNSCC

**Figure 3:**
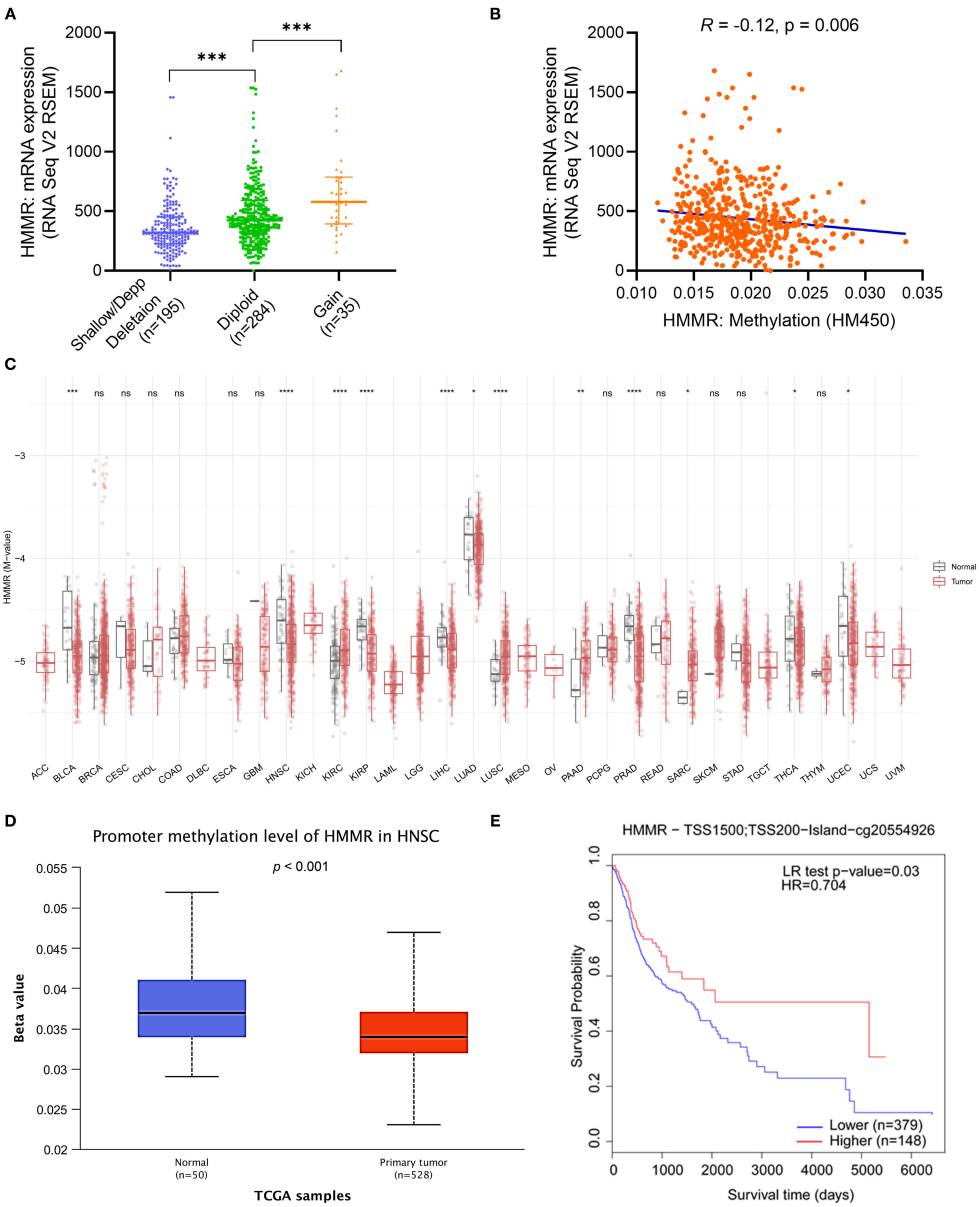
The copy number variation (CNV) and methylation of HMMR in HNSCC. **(A)** the expression level in different CNV of HMMR (*n* = 514); **(B)** the correlation between HMMR methylation and its expression level (*n* = 521); **(C)** The methylation levels of HMMR in pan-cancer and normal tissues from TCGA data; **(D)** The promoter methylation of HMMR in tumor tissues (*n* = 528) and normal tissues (*n* = 50) from TCGA-HNSCC data; **(E)** the Kaplan–Meier survival of the promoter methylation of HMMR in HNSCC (*n* = 527). ^*^p < 0.05, ^**^p < 0.01, ^***^p < 0.001, ^****^p < 0.0001.

After verifying the prognosis of HMMR, we used cBioPortal to analyze the association of mRNA expression of HMMR and its copy number variation (CNV) and methylation data in HNSCC. It was found that the amplification of CNV in HMMR was not observed. Patients with the gain of CNV of HMMR had a higher level of HMMR expression in HNSCC, but only 6.8 % of patients (35/514) exhibited this (Figure 3A). This suggests that CNV may not be the main cause behind the high expression of HMMR. We further analyzed the relationship between *HMMR* methylation and gene expression, and these results showed that gene methylation negatively correlated with HMMR gene expression (*R* = −0.12, *p* < 0.001) (Figure 3B). The methylation levels of *HMMR* were significantly lower in a variety of tumors than in normal tissues from TCGA database, including BLCA, HNSC, KIRC, KRPA, LIHC, LUAD, PRAD, thyroid carcinoma (THCA) and UCEC (Figure 3C). The promoter methylation of *HMMR* in tumor tissues of TCGA-HNSCC was significantly lower than that of normal tissues adjacent to the cancer in UALCAN webpage (*p* < 0.001; Figure 3D). In addition, the MethSurv analyses showed that patients with low *HMMR* methylation had a worse OS than patients with high methylation (*p* < 0.001; Figure 3E).

### Functional Enrichment Analysis of High- and Low-*HMMR* Expression Samples

**Figure 4:**
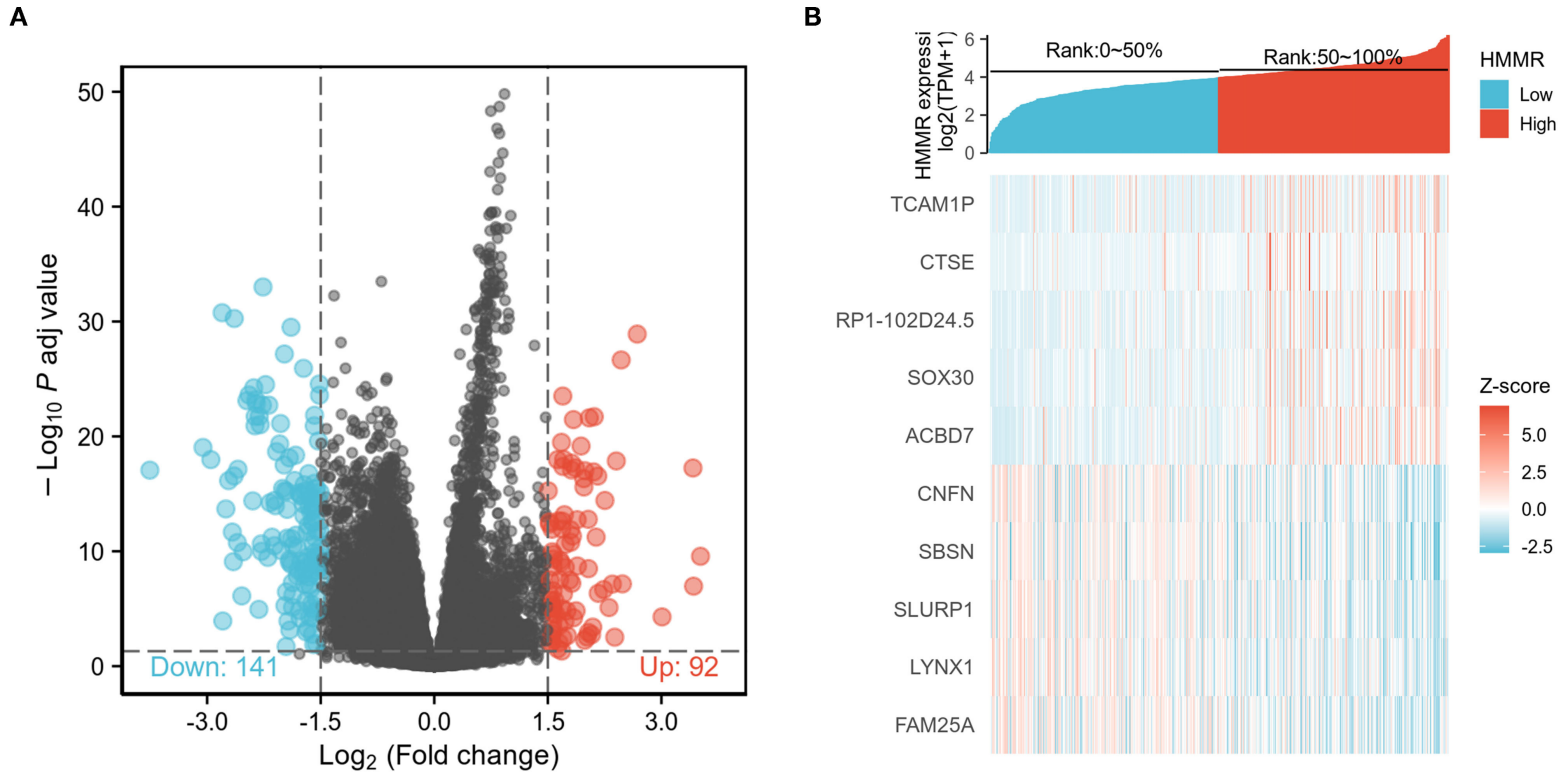
**(A)** Volcano Plot of differentially expressed genes (DEGs); **(B)** Heatmap of differentially expressed genes (DEGs).

**Figure 5:**
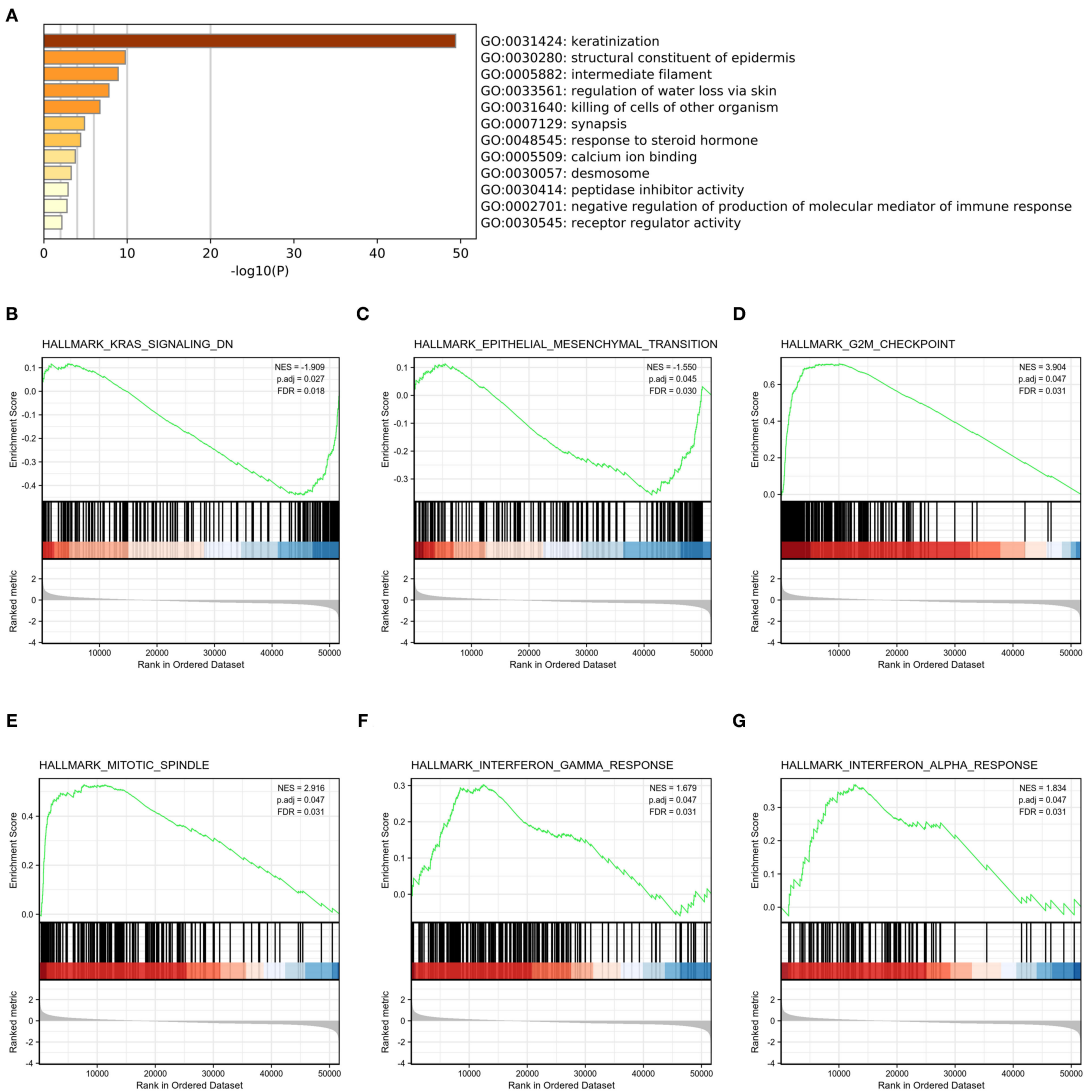
Functional enrichment of HMMR in HNSCC. **(A)** GO enrichment analysis of differentially expressed genes (DEGs) in high- and low-HMMR expression samples; **(B)** Enrichment of genes in the KRAS signaling pathway by GSEA; **(C)** Enrichment of genes in the EMT signaling pathway by GSEA; **(D)** Enrichment of genes in the G2M checkpoint pathway by GSEA; **(E)** Enrichment of genes in the Mitotic spindle pathway by GSEA; **(F)** Enrichment of genes in the interferon-gamma response by GSEA; **(G)** Enrichment of genes in the interferon-alpha response by GSEA.

To explore the potential mechanisms of HMMR that promote tumor progression, we analyzed DEGs in the high- and low-*HMMR* expression samples. A total of 233 DEGs were identified, of which 92 genes were upregulated, and 141 were downregulated. The DEGs's expression is shown in a heatmap and Volcano Plot (Figures 4A,B). Following this, the functions of co-expression in patients with HNSCC were predicted using GO enrichment analysis. The top GO enrichment items in the biological process (BP), molecular function (MF), and cellular component (CC) groups were keratinization, structural constituent of epidermis, intermediate filament, regulation of water loss via skin, synapsis, response to steroid hormone, calcium ion binding, desmosome, peptidase inhibitor activity, negative regulation of production of molecular mediator of immune response, and receptor regulator activity (Figure 5A). We also performed a GSEA analysis to identify the key pathways related to *HMMR*. GSEA analysis found that 19 data sets satisfied the criteria of an FDR < 0.25 and a *p* < 0.05, shown in Table 4. The most significantly enriched pathways were KRAS signaling (Figure 5B), epithelial-mesenchymal transition (EMT) (Figure 5C), G2M checkpoint (Figure 5D), mitotic spindle (Figure 5E), interferon-gamma response (Figure 5F), and interferon-alpha response (Figure 5G).

**Table 4:** Hallmark Pathways enriched in high- and low-risk groups by using GSEA.

**ID**	**NES**	**p value**	**p.adjust**	**FDR**
HALLMARK_KRAS_SIGNALING_DN	−1.909	0.001	0.027	0.018
HALLMARK_MYOGENESIS	−2.822	0.001	0.027	0.018
HALLMARK_PANCREAS_BETA_CELLS	1.692	0.004	0.045	0.03
HALLMARK_EPITHELIAL_MESENCHYMAL_TRANSITION	−1.55	0.004	0.045	0.03
HALLMARK_MYC_TARGETS_V2	1.722	0.005	0.045	0.03
HALLMARK_SPERMATOGENESIS	2.529	0.009	0.047	0.031
HALLMARK_E2F_TARGETS	4.224	0.012	0.047	0.031
HALLMARK_G2M_CHECKPOINT	3.904	0.012	0.047	0.031
HALLMARK_INTERFERON_GAMMA_RESPONSE	1.679	0.012	0.047	0.031
HALLMARK_MTORC1_SIGNALING	1.524	0.012	0.047	0.031
HALLMARK_MYC_TARGETS_V1	1.971	0.012	0.047	0.031
HALLMARK_MITOTIC_SPINDLE	2.916	0.012	0.047	0.031
HALLMARK_INTERFERON_ALPHA_RESPONSE	1.834	0.012	0.047	0.031
HALLMARK_INFLAMMATORY_RESPONSE	1.268	0.024	0.084	0.055
HALLMARK_ANDROGEN_RESPONSE	1.51	0.025	0.084	0.055
HALLMARK_APICAL_SURFACE	−1.518	0.034	0.105	0.069
HALLMARK_ALLOGRAFT_REJECTION	1.248	0.036	0.105	0.069
HALLMARK_P53_PATHWAY	−1.331	0.044	0.12	0.079
HALLMARK_IL6_JAK_STAT3_SIGNALING	1.396	0.046	0.12	0.079

*GSEA, gene set enrichment analysis; NES, normalized enrichment score; FDR, false discovery rate*.

### The Correlation Between *HMMR* Expression and the Infiltration of Immune Cells

**Figure 6:**
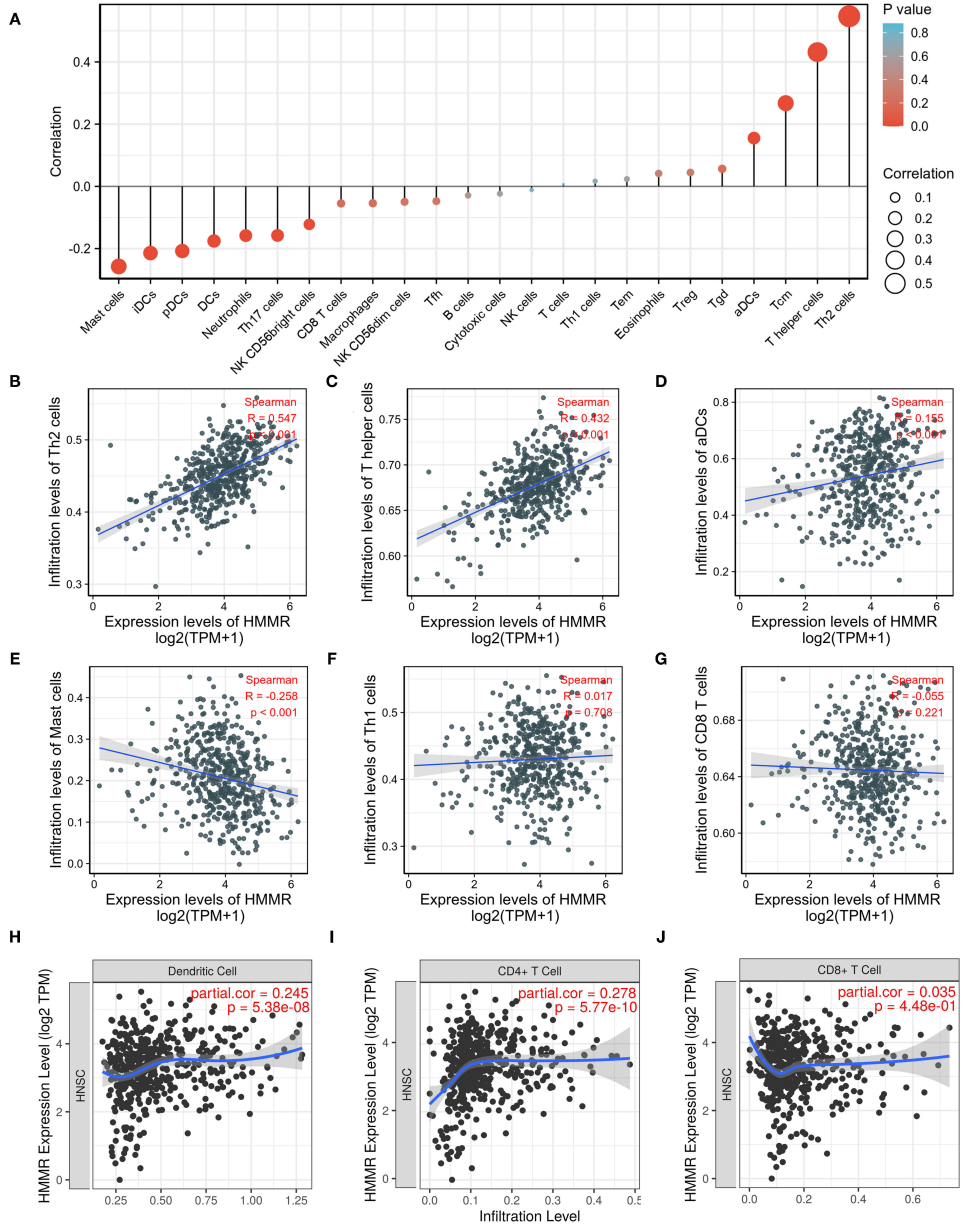
ssGSEA analyses of HMMR and the correlation of HMMR expression with immune infiltration level in HNSCC. **(A)** The correlation between the infiltration of immune cells and the expression of HMMR; **(B–D)** HMMR expression significantly positively correlates with infiltrating levels of Th2 cells **(B)**, T helper cells **(C)**, and activated dendritic cells **(D)**; **(E)** HMMR expression significantly negatively correlates with infiltrating levels of master cells; **(F,G)** HMMR expression has no correlations with infiltrating levels of The 1 cells **(F)** and CD8+ T cells **(G)**; **(H–J)** HMMR expression significantly positively correlates with infiltrating levels of dendritic cells **(H)**, and CD4+ T cells **(I)**, but not CD8+ T cells **(J)**.

Considering that both GO and GSEA enrichment analysis found that the HMMR may participate in the tumor immune response, we further applied ssGSEA to analyze the relationship between the *HMMR* mRNA expression and the infiltration level of immune cells. The correlation between the infiltration of immune cells and the *HMMR* mRNA expression is shown in Figure 6A. The results indicated that the *HMMR* mRNA expression positively correlated with the infiltration of Th2 cells (*R* = 0.551, *p* < 0.001, Figure 6B), T helper cells (*R* = 0.497, *p* < 0.001, Figure 6C) and activated dendritic cells (DCs) (*R* = 0.174, *p* < 0.001, Figure 6D). ssGSEA also showed that HMMR expression negatively correlated with the infiltration of Mast cells (*R* = −0.218, *p* < 0.001, Figure 6E), while the *HMMR* mRNA expression did not correlate with CD8+ T cells (*R* = −0.035, *p* = 0.437, Figure 6F) and Th1 cells (*R* = 0.048, *p* = 0.284, Figure 6G). Furthermore, the analyses by TIMER software showed that the *HMMR* mRNA expression positively correlated with the infiltration of DCs (*R* = 0.245, *p* < 0.001, Figure 6H) and CD4+ cells (*R* = 0.278, *p* < 0.001, Figure 6I), but not with the infiltration of CD8+ T cells (*R* = 0.035, *p* = 0.448, Figure 6J).

### Validation the HMMR Expression and Prognostic Value of HMMR in HNSCC

**Figure 7:**
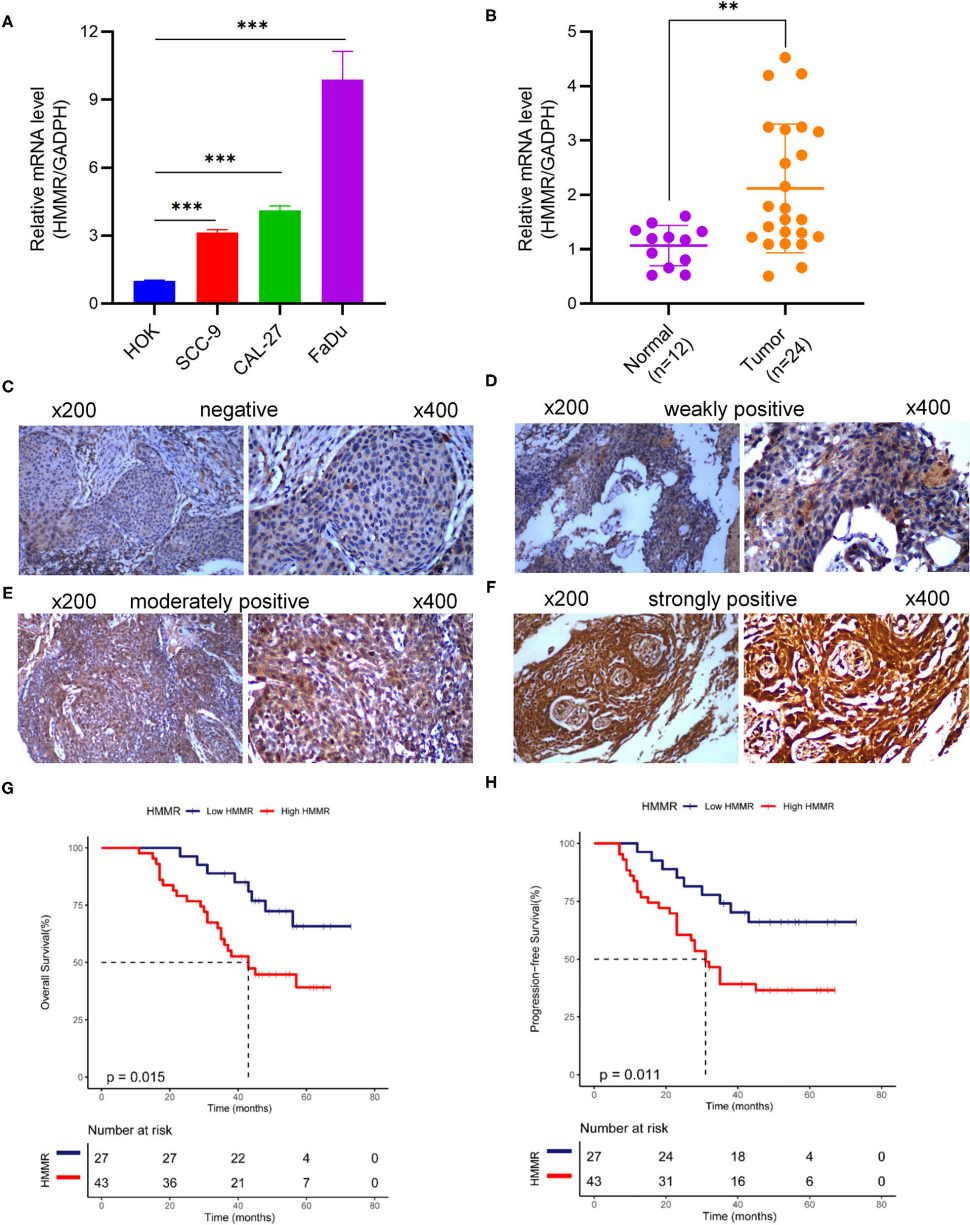
HMMR mRNA and protein expression in cell lines and HNSCC tissues. **(A)** HMMR mRNA expression in normal human oral keratinocyte and three HNSCC cell lines; **(B)** HMMR mRNA expression in normal tissue (*n* = 12) and HNSCC tissues (*n* = 24); **(C–F)** representative IHC staining patterns of HMMR in HNSCC tissues: **(C)** negative, **(D)** weakly positive, **(E)** moderately positive, and **(F)** strongly positive; **(G)** Overall survival rate of HMMR in HNSCC (*n* = 70); **(H)** Progression free survival of HMMR in HNSCC (*n* = 70). ^**^p < 0.01, ^***^p < 0.001.

The results showed that at the cellular level, the *HMMR* mRNA expression in HNSCC cell lines (SCC9, CAL27, and FaDu) was significantly higher than that of HOK (all *p* < 0.001; Figure 7A). The expression of HMMR in HNSCC tissues was also significantly higher than that in normal adjacent tissues (*p* < 0.001; Figure 7B). At the same time, IHC was used to detect *HMMR* protein expression in HNSCC tissues. Among 70 HNSCC cases, 14 cases were negative, 13 cases were weakly positive, 39 were moderately positive, and 4 were strongly positive. The overall positive rate was 80.0%, and only 14 cases (20.0%) were *HMMR*-negative. The median follow-up time was 55 months (range 11–73 months). Detailed demographic and clinical characteristics are listed in Table 5. Based on *HMMR* protein expression in the tumor tissues, HNSCC patients were divided into low (negative and weekly positive) and high (moderately and strongly positive) expression groups. Kaplan–Meier survival analyses showed that patients with high *HMMR* protein expression had a lower 5-years OS (39.2 vs. 65.8%, *p* = 0.015) and PFS (36.6 vs. 66.0%, *p* = 0.011) than those with low *HMMR* expression (Figures 7G,H). MAV confirmed that high *HMMR* protein expression was an inferior independent factor for OS (HR = 2.288, 95% CI: 1.019–5.137, *p* = 0.045) and PFS (HR = 2.247, 95% CI: 1.048–4.817, *p* = 0.038) when adjusted for gender, age, clinical stage, histologic grade and Anatomic site (Table 6).

**Table 5:** Demographic and clinical characteristics of HNSCC patients with low- and high-expression HMMR in protein level (*n* = 70).

**Characters**	**level**	**HMMR expression**		***p***
		**Low (*n* = 27)**	**High (*n* = 43)**	
Gender	Female	2 (7.4%)	3 (7.0%)	0.999
	Male	25 (92.6%)	40 (93.0%)	
Age	<60 years	15 (55.6%)	17 (39.5%)	0.685
	≥60 years	12 (44.4%)	26 (60.5%)	
T classification	T1–T2	13 (48.1%)	17 (39.5%)	0.478
	T3–T4	14 (51.9%)	26 (60.5%)	
N classification	N0–1	24 (88.9%)	31 (72.1%)	0.137
	N2–3	3 (11.1%)	12 (27.9%)	
Clinical stage	Stage I–II	12 (44.4%)	14 (32.6%)	0.316
	Stage III–IV	15 (55.6%)	29 (67.4%)	
Histologic grade	G1	16 (59.3%)	13 (30.2%)	0.055
	G2	6 (22.2%)	15 (34.9%)	
	G3	6 (18.5%)	15 (34.9%)	
Anatomic site	Oral cavity	8 (29.6%)	7 (16.3%)	0.549
	Oropharynx	1 (3.7%)	3 (7.0%)	
	Hypopharynx	1 (3.7%)	3 (7.0%)	
	Larynx	17 (63.0%)	30 (67.8%)	

**Table 6:** The multivariate analyses of overall survival and progression-free survival according to HMMR protein expression, after adjusting for other potential predictors (*n* = 70).

**Characteristics**	**Overall survival**		**Multivariate analysis**	
	**HR (95% CI)**	***P-*value**	**HR (95% CI)**	***P-*value**
Clinical stage (I–IIvs. III–IV)	2.644 (0.966–7.234)	0.058	3.109 (1.157–8.353)	0.024
Age (≤ 60 vs. >60)	1.270 (0.600–2.689)	0.533	1.296 (0.635–2.647)	0.476
Gender (Female vs. Male)	0.754 (0.076–7.516)	0.810	0.688 (0.071–6.646)	0.747
Histologic grade (G1 vs. G2/G3)	1.075 (0.514–2.249)	0.848	1.134 (0.559–2.298)	0.728
Anatomic site (Oral cavity/Oropharynx/Hypopharynx vs. Larynx)	2.049 (0.782–5.371)	0.144	1.949 (0.802–4.737)	0.141
HMMR (Low vs. High)	2.288 (1.019–5.137)	0.045	2.247 (1.048–4.817)	0.038

## Discussion

Considering the strong heterogeneity of HNSCC and its unsatisfactory OS rate (3, 4), it is important to effectively and accurately assess the prognosis of HNSCC. This study found that HMMR is significantly overexpressed in HNSCC, and its overexpression is associated with HMMR hypomethylation. Furthermore, *HMMR* mRNA expression, protein expression, and hypomethylation of *HMMR* were both associated with adverse OS in HNSCC. Functional enrichment analysis found that *HMMR* expression was related to KRAS signaling, EMT, G2M checkpoint, Mitotic spindle, interferon-gamma, and interferon-alpha responses, and increased the infiltration level of Th2 cells. Thus, our study provides insights into understanding the potential role of HMMR in tumor pathogenesis and demonstrates its use as a potential HNSCC biomarker.

This study showed that *HMMR* is highly expressed in HNSCC compared with normal tissues (*p* < 0.001) from data in the TCGA database and our specimens. This result is consistent with previous studies that have also found the *HMMR* protein to be highly expressed in various types of cancer, including bladder cancer (13), pancreatic cancer (14, 15), glioma (16), gastric cancer (17), and colorectal cancer (18). Our analysis corroborated this as we found that *HMMR* is significantly overexpressed in most tumors in TCGA data. These results suggest that HMMR has the potential to become a diagnostic marker in various cancers. In our analysis, the expression of HMMR was a good diagnostic marker in HNSCC, and its AUC exceeded 0.9. In addition, we also found that *HMMR* is related to the N classification, clinical stage and pathological grade of HNSCC, further supporting that the expression of *HMMR* may be related to the degree of malignancy of HNSCC. *HMMR* is considered to be a tumor-associated antigen (21); previous reports have shown that HMMR can be secreted extracellularly (21, 34). Recent studies have found that *HMMR* expression can be detected in patients' urine using enzyme-linked immunosorbent assay (ELISA) (35), which suggests that *HMMR* expression could be a convenient diagnostic biomarker in a variety of tumors, including HNSCC.

*HMMR* is highly expressed in HNSCC and is related to poor prognosis. Our study found that patients with a high HMMR mRNA expression, from TCGA HNSCC data, had a worse OS, and which was an independent prognostic factor of OS and PFS. This result was also verified in HNSCC data from the GEO data set (GSE41613) as multivariate analysis also found that *HMMR* is an independent prognostic factor for HNSCC. Besides, our study showed that *HMMR* protein expression was a prognostic biomarker in HNSCC. Previously, Shigeishi et al. reported that the OS of HNSCC patients with high expression of *HMMR* protein was worse than those with low expression, but the difference was not statistically significant. The main reason is that the number of patients tested by immunohistochemistry is too small, just only 35 patients (26). Although this is the first study to show that *HMMR* expression could be a prognostic factor for HNSCC, a large number of relevant studies have revealed that *HMMR* expression could be an adverse prognostic biomarker for various tumors (14–18). Considering that HMMR is a strong prognostic factor, we constructed a nomogram combining the *HMMR* expression and clinical data. The nomogram more accurately predicted the 3- and 5-year OS for patients with HNSCC. The nomogram could help to screen for high-risk patients and determine more aggressive treatment regimens for high-risk patients with HNSCC. We also explored the mechanism of *HMMR* mRNA overexpression in HNSCC, and our results showed that HMMR overexpression might be related to HMMR hypomethylation. Interestingly, HMMR methylation was adverse associated with the prognosis of HNSCC, and hypomethylated patients have worse OS, which is consistent with the prognostic value of the mRNA expression of this gene. Although many mechanisms can give rise to elevated gene expression, hypomethylation is one of the main regulatory mechanisms of gene expression.

As a HA receptor, *HMMR* can play a role in a variety of biological functions that lead to the development of tumors (36). Enrichment analysis found that *HMMR* may participate in the KARS signaling pathway, EMT, and other pathways in HNSCC, which is also consistent with previous research. Previous studies have found that *HMMR* may be involved in multiple biological functions such as cell proliferation, cycle regulation, migration, and invasion (36–38). Interestingly, GSEA found that *HMMR* may be related to the interferon response pathway. Furthermore, ssGSEA also showed that HMMR positively correlated with the infiltration level of Th2 cell, but did not correlate with CD8+T and DC cell infiltration. Previous studies have found that the infiltration of Th2 cell is associated with immunosuppression and poor survival in a variety of tumors (39–41). In this study, Th2 cells were significantly increased, which suggests that HMMR may help to mediate the immune escape of HNSCC. A similar situation occurs with the tumor-associated antigen EpCAM, which promotes Th2 cell-mediated immune escape (40).

Our study found that *HMMR* expression is increased in HNSCC, and high expression was a factor for poor prognosis. This suggests that *HMMR* could be a therapeutic target for HNSCC. Considering that *HMMR* can be considered a tumor-associated antigen, immunotherapy with the *HMMR* protein could be a novel strategy. Previous studies have shown that short peptide HMMR can be effectively presented by DC cells, activate T cell immunity, and induced positive immunological responses (19–22). In addition, T-cell receptor-engineered T-cell therapy of *HMMR* efficiently inhibitor tumor growth in an animal model (23). However, TCR-T treatment requires MHC I pairing, making it a challenging and complex treatment strategy. Considering that *HMMR* can be expressed on the tumor surface, CAR-T targeting of HMMR could also be a future treatment strategy. MacKay et al. (42) considered HMMR as a potential target for CAR-T therapy, but this still requires further research. Therefore, HMMR has value as a possible immunotherapy target in the future.

Although this study improved our understanding of the relationship between HMMR and HNSCC, there were some limitations. Firstly, our result cannot be validated due to the absence of *in vitro* and *in vivo* experience. Furthermore, due to the design's limitations of our study, additional critical signaling pathways associated with *HMMR* may have been missed, and these relevant pathways should be examined further. To further investigate the mechanism of *HMMR* in the HNSCC, we have cultivated cell lines for wet lab work in the near future.

In conclusion, *HMMR* mRNA expression was overexpressed in HNSCC, while methylation of HMMR was decreased in HNSCC. Moreover, a high *HMMR* mRNA and protein expression and hypomethylation level both related to poor OS. Enrichment analysis indicated that *HMMR* might act as an oncogenic factor by regulating the EMT of tumor cells and suppressing adaptive immunity by promoting the infiltration of Th2 cells. This study demonstrated *HMMR* as a potential biomarker for diagnosis and prognosis of HNSCC, highlighting it as a potential immunotherapy target.

## Data Availability Statement

The datasets presented in this study can be found in online repositories. The names of the repository/repositories and accession number(s) can be found in the article/Supplementary Material.

## Author Contributions

TL, XG, QG, and JL participated in the conception and design of the study. YZ, JC, and QL performed the experiments. ZT, JP, SL, and JL interpreted the data produced and edited the drafts of the manuscript. All authors read and approved the final manuscript.

## Conflict of Interest

The authors declare that the research was conducted in the absence of any commercial or financial relationships that could be construed as a potential conflict of interest.

## Supplementary Material

Supplementary Figure 1 Survival curves of Progression-free Survival from TCGA-HNSCC data (*n* = 500).

Supplementary Figure 2 The prognostic value of HMMR expression in HNSCC excepted for HPV (+) OPC and HPV (+) OPC from TCGA-HNSCC data. **(A)** Survival curves of Overall Survival for HNSCC excepted for HPV (+) OPC from TCGA-HNSCC data (*n* = 453); **(B)** Survival curves of Overall Survival for HPV (+) OPC from TCGA-HNSCC data (*n* = 47).

Supplementary Table 1 The univariate and multivariate analyses of Progression-free Survival according to HMMR expression, after adjusting for other potential predictors in TCGA (*n* = 500).

## Abbreviations

HNSCC: Head and neck squamous cell carcinoma
OS: Overall survival
HMMR: hyaluronan-mediated motility receptor
TCGA: The Cancer Genome Atlas database
GEO: Gene Expression Omnibus
GO: Gene ontology
HPV: human papillomavirus
ECM: extracellular matrix
CNV: copy number variation
ROC: Receiver operating characteristic
EMT: mesenchymal transition.
