# Supplementary figures and images for: High Expression of Hyaluronan-Mediated Motility Receptor Predicts Adverse Outcomes: A Potential Therapeutic Target for Head and Neck Squamous Cell Carcinoma
Source: Front Oncol. 2021 Mar 8;11:608842. doi: 10.3389/fonc.2021.608842 (PMC7982417; doi:10.3389/fonc.2021.608842)

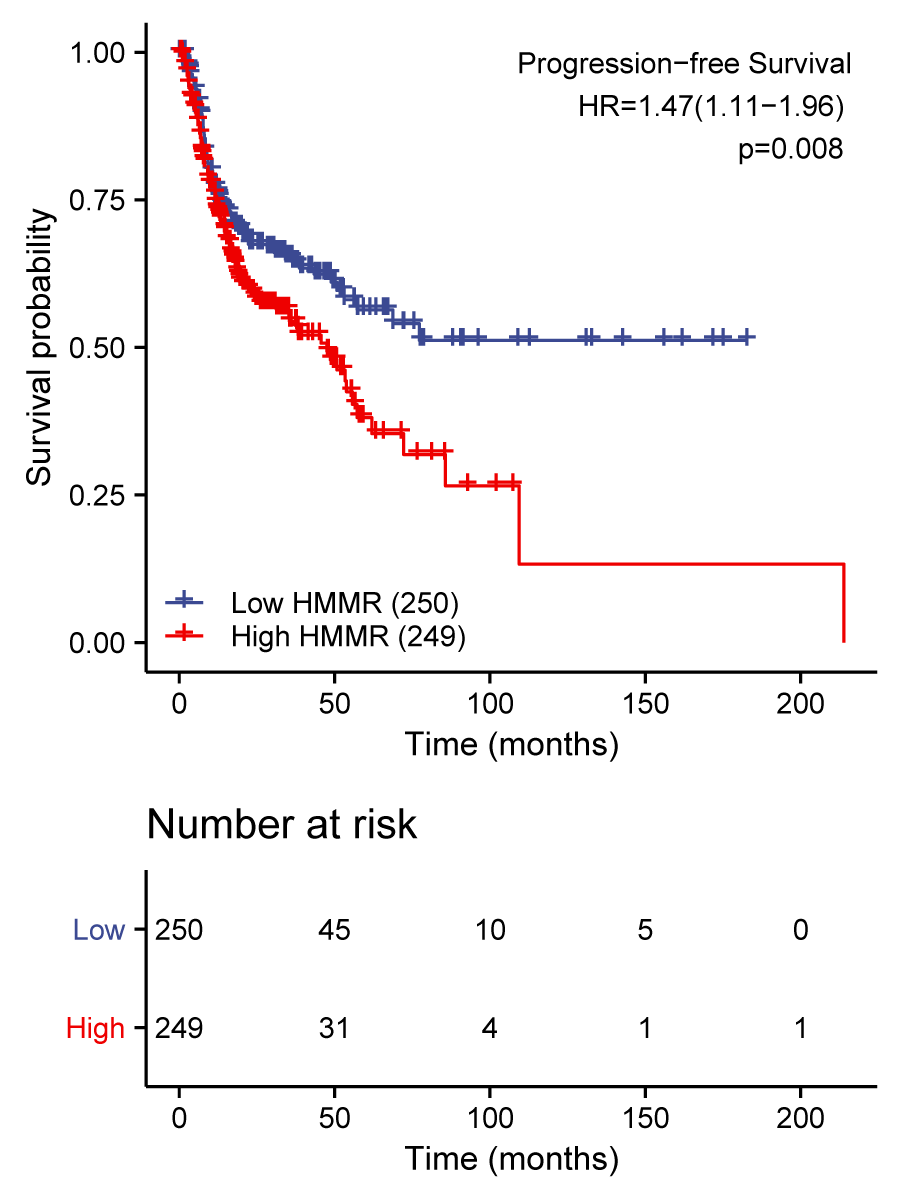

Supplement: Supplementary Figure 1 — Survival curves of Progression-free Survival from TCGA-HNSCC data (n = 500). [file Image_1.TIF]

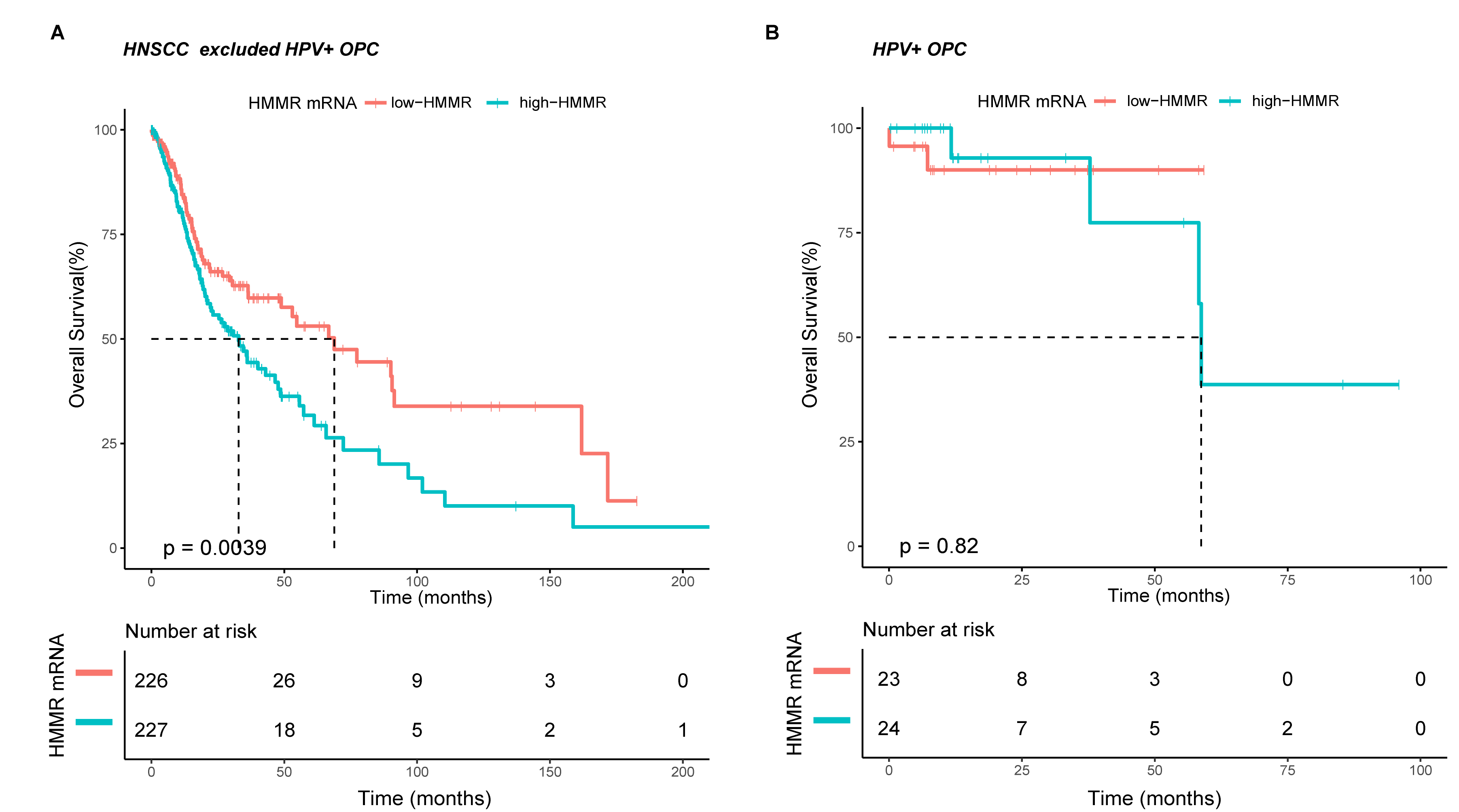

Supplement: Supplementary Figure 2 — The prognostic value of HMMR expression in HNSCC excepted for HPV (+) OPC and HPV (+) OPC from TCGA-HNSCC data. (A) Survival curves of Overall Survival for HNSCC excepted for HPV (+) OPC from TCGA-HNSCC data (n = 453); (B) Survival curves of Overall Survival for HPV (+) OPC from TCGA-HNSCC data (n = 47). [file Image_2.TIF]
