# Supplementary material for: High Expression of Hyaluronan-Mediated Motility Receptor Predicts Adverse Outcomes: A Potential Therapeutic Target for Head and Neck Squamous Cell Carcinoma
Source: Front Oncol. 2021 Mar 8;11:608842. doi: 10.3389/fonc.2021.608842 (PMC7982417; doi:10.3389/fonc.2021.608842)
Supplement: Supplementary Table 1 — The univariate and multivariate analyses of Progression-free Survival according to HMMR expression, after adjusting for other potential predictors in TCGA (n = 500). [file Table_1.docx]

**Supplementary Table 1. The univariate and multivariate analyses of Progression-free Survival according to HMMR expression, after adjusting for other potential predictors in TCGA.**

| Characteristics | Univariate analysis | |  | Multivariate analysis | |
| --- | --- | --- | --- | --- | --- |
|  | HR(95% CI) | P value |  | HR(95% CI) | P value |
| T stage (T1-2 vs. T3-4) | 0.755(0.553-1.032) | 0.078 |  | 0.758(0.548-1.047) | 0.092 |
| N stage (N0 vs. N1-3) | 0.799(0.597-1.068) | 0.130 |  |  |  |
| M stage (M1 vs. M0) | 0.352(0.112-1.106) | 0.074 |  | 0.272(0.086-0.862) | 0.027 |
| Clinical stage ( I-II vs. III- IV) | 0.842(0.592-1.195) | 0.335 |  |  |  |
| Age (≤60 vs. >60) | 1.063(0.800-1.412) | 0.674 |  |  |  |
| Gender (Female vs. Male) | 1.042(0.753-1.442) | 0.803 |  |  |  |
| Histologic grade (G1-2 vs. G3-4 | 0.973(0.702-1.351) | 0.872 |  |  |  |
| Smoker (No vs. Yes) | 0.888(0.639-1.235) | 0.481 |  |  |  |
| Alcohol history (No vs. Yes) | 1.400(1.014-1.932) | 0.041 |  | 1.386(0.990-1.940) | 0.057 |
| TP53 status (WT vs. Mut) | 1.366(0.993-1.879) | 0.055 |  | 1.425(1.017-1.997) | 0.040 |
| PIK3CA status (WT vs. Mut) | 1.043(0.722-1.506) | 0.824 |  |  |  |
| Radiation therapy (No vs. Yes) | 0.854(0.616-1.183) | 0.342 |  |  |  |
| Race (White vs. Non-white) | 1.416(0.921-2.177) | 0.113 |  |  |  |
| HPV (Negative vs. Positive) | 1.335(0.894-1.995) | 0.158 |  |  |  |
| HMMR (Low vs. High) | 1.473(1.106-1.961) | 0.008 |  | 1.535(1.138-2.071) | 0.005 |
